# Books and Authors

**Published:** 1906-10

**Authors:** 


### BOOKS AND AUTHORS.

**Christianity and Sex Problems.** By **Hugh Northcote,** M.A. Crown Octavo, 257 pages. F. A. Davis Company, Philadelphia, Pa. (Price $2.00 net).

This is an alluring mixture of topics and one which needs much careful thought to get at the heart of the subject, for while visible sociologic conditions draw us to the belief that christianity and sex do mix well on the surface, experience tends to lead us to a more or less unwelcome knowledge that sex can no more be controlled by pure christianity than can disease. There is fascination in this delving into the whys and wherefores of sex problems, and it attracts the religious mind quite as irresistably as it does the more calculating medical man's attention.

Viewed from a purely christian standpoint and gazed at through the usual long range glasses from the pulpit and handled with gloves as nearly all who are not closely associated with sexual matters handle such questions, pathologic or sentimental, christianity, it must be confessed, does present a purely theoretic solution to all the troubles which beset society. Yet, as a cure-all for sexual problems as is indicated in this book, christianity is a failure and a farce. The great trouble with works dealing with sex from the Bible standpoint; or let us say, from the standpoint of the christian, the man who believes that in chrisianity alone lies the solution, is that the question is merely skimmed over and there is invariably a daintiness in the handling of the problems which does not carry conviction, and shows a too highly developed sense of maidenly modesty. To adequately present a subject in book form an author must get down to the level of what he is writing about; he must know it, not merely understand it, and to know it he must be familiar with it in all its phases and recognise it from every angle. More, he must associate himself with it and not be merely content to discuss what others have learned.

As a book of reference this one is of value, the foot notes drawing attention to the writings of others and the biblic references to sex being most complete. As a whole the book is a reflection of quite all the serviceable and some of the idiotic writings on this very complex matter of sex. The author's comments on the statements of Ellis, Westermark and other authorities are interesting, and the fact that he is in accord with the larger part of their teachings gives evidence of the receptivity of his mind and shows him to be anything but narrow or bigoted. The statement credited to Juvenal, however, that immorality in children "is not so much a product of conscious and wilful depravity as of hard and strained conditions of life," can hardly be accepted by those who are at all familiar with sex study outside the confines of the mission room or the experience meeting. There is conscious depravity in children and it is not due to the hard and strained conditions of life. One must admit this when he hears a group of girls ranging from 11 to 16 brazenly confess their habitual prostitution for money, and glorying in it. There is something more than strained conditions of life to account for it.

In that section devoted to alcoholism and houses of prostitution the author apparently has laid too much reliance on the unsupported statements of the amateur mission workers—people of notoriously narrow mind and broad imaginations. Were it not so amusing it would be pathetic to see set forth in type in a book on sexual matters the statement ascribed to a reformed prostitute, that "nearly every man" entering one of these lidded palaces of pleasure was intoxicated, the inference being that he knew not what he did or where he went. Yet, that is merely an allusion and not an opening for a discussion of the "social evil" as we are happy to term prostitution when we discuss it among our church societies, and when we write horror-stricken and foolish letters to the newspapers. In this he is wise. But, when the statement is made that 14% of boys have varicocele which has been "contracted" there is reason for much wonder, and again the struggle between the ludicrous and the pathetic is presented. Like nearly all works which endeavor to set up christianity as the one hope of a sin-besodden world, there is too much sentiment and too little sense; too much trimmings and not enough substance; too many illustrations which are patently exaggerated, not by the author necessarily, but by those from whom he has received the facts in his investigations. Here, for instance is the tale of a boy, a nice clean, gentlemanly boy with high ideals and pure mind, who was just as nice a boy as a boy could very well be and still remain human. One day in school he saw another boy flogged—and then he went to a brothel. And the horrible example is closed by the author with this thrilling statement: "The moral rot which wrought such disaster to a once promising career set in as the direct result of the ill-omened contact with prostitution." Sad, isn't it?

In the handling of masturbation it does not seem possible that the writer can be sincere in his concurrence with the views of some of the top-heavy maiden lady writers on boyhood and sex whom he quotes, for it is recommended to mothers that they can cure the habit by praying for their son's welfare and strength, and that a most excellent procedure is to sit by the bedside when the child has fallen asleep, take his hand in theirs and make low-voiced suggestion that the habit is to be broken. One might imagine that this suggestion came from the eminent Mary Baker instead of a woman whose weight of ideas might fit her for a place among the bassos in a choir.

Briefly, there are many excellent thoughts in the book. The author has gone into the subject very thoroughly and his list of references is invaluable. Yet, it must be confessed that in addition to all the good in the book there is an unmistakable flavor of cant; but one must expect that in any work which, while recognising sex and sex problems, throws about them the banner of the church and declares in substance that the narrow aisle approaching the altar is the only road to solution and salvation. It's fine in theory, but it doesn't work out in practical experience.

Christianity does much to prepare the world for the battle with the problems—it can do much more. But it will never become a uniformly successful therapeutic measure. It's field is rather prophylactic than otherwise and it has got to be dealt out with judicious hands.

**State Commission in Lunacy for the State of New York.** Sixteenth Annual Report, October 1, 1903 to September 30, 1904. Transmitted to the Legislature February 8, 1905. From Hon. Henry W. Hill, Senator 47th District. Albany: Brandow Printing Company. 1905.

The annual reports of this commission contain interesting facts relating to the insane in this state. During the period covered by this report the cost of maintenance was $5,161,198.06, the total number of patients treated being 26,861. The average number under daily care was 24,647.

Included in the report is a detailed statement as to expenses in each hospital and an elaborate statement by the pathologist. Tribute is paid to Dr. Peterson who resigned May 14, 1904, and general medical affairs of the hospital receive appropriate attention. The book contains many statistical tables, and other items of interest to the political economist who gives attention to the insane.

---

**A Treatise on Surgery.** By **George Ryerson Fowler,** M.D., Examiner in Surgery, Board of Medical Examiners of the Regents of the University of the State of New York; Emeritus Professor of Surgery in the New York Polyclinic, etc. Two imperial octavos of 725 pages each, with 888 text illustrations and 4 colored plates. all original. Vol. II. Philadelphia and London: W. B, Saunders Company, 1906. (Per set: cloth, $15.00, net; half-morocco, $17.00, net).

The second volume of this comprehensive treatise fully sustains the opinion expressed by the journal in commenting upon the first—namely, that Fowler's surgery is the most substantial contribution to the textbook literature of the subject in recent years. It represents the patient study of a practical and resourceful surgeon during twelve years of mature life, when his activities and judgment were at their best. It is the crowning effort of a ripened manhood that was always reaching out for something better—and obtained it.

This volume continues the topic of regional surgery beginning with that of the dorsal and lumbar vertebræ, then taking up in regular order, surgery of the abdominal and pelvic regions; surgery of the female pelvic organs; surgery of the upper extremity; and surgery of the lower extremity.

As might be anticipated with reason, appendicitis receives considerable attention, it being the subject of a special treatise or monograph written by Fowler five or six years ago. It was, alas! the very irony of fate that he should succumb to the disease in the treatment of which he was an expert, and upon the entire details of which he was an acknowledged authority. Hernia is another topic that is elaborately discussed and much light is thrown upon this always attractive subject. The kidneys form another interesting field for this author, who presents many suggestions of value in regard to it.

Fowler was an ingenious surgeon who offered many new points in technic for the consideration of his colleagues. Among these may be mentioned Fowler's position, (the reversed Trendelnberg) ; Fowler's incision in appendecitis; Fowler's infolding intestinal suture; Fowler's method of colopexy in prolapse of the rectum; Fowler's operation for hemorrhoids; Fowler's operation for prolapse of the rectum; Fowler's operation for retrodeviation of the uterus; Fowler's operation for syndactylism, and Fowler's universal needle forceps.

The treatise is profusely illustrated with original figures and drawings in the text and four colored plates. It is a distinct addition to the surgical literature of the period and is unsurpassed by any work that has yet appeared, whether desired by the specially trained surgeon or the general practitioner.

